# Allo-Hemodialysis: A Narrative Review of a Novel Approach to Resource-Sparing Kidney Replacement Therapy

**DOI:** 10.3390/jcm15218717

**Published:** 2026-11-08

**Authors:** Peter Kotanko, Vaibhav Maheshwari, Xin Wang, Nadja Grobe, Alhaji Cherif, Xia Tao, Rasha Hussein, Amrish Patel, Joshua Emmanuel Chao, Alexander Heide, Dejan Nikolic, Jiaming Dong, Guido Filler, Maria Díaz-González de Ferris, Nicholas Steneck, Karopadi Shivanand Nayak

**Affiliations:** 1 Renal Research Institute, New York, NY 10065, USAnadja.grobe@rriny.com (N.G.);; 2 Icahn School of Medicine at Mount Sinai, New York, NY 10029, USA; 3 Department of Pediatric Nephrology, Soba University Hospital, Khartoum 13314, Sudan; 4 Fresenius Medical Care Deutschland GmbH, 97424 Schweinfurt, Germany; 5 Department of Paediatrics, Western University, London, ON N6A 3K7, Canada; guido.filler@lhsc.on.ca; 6 School of Medicine at Chapel Hill, The University of North Carolina, Chapel Hill, NC 25799, USA; 7 Department of History, University of Michigan, Ann Arbor, MI 48109, USA; 8 Virinchi Hospital, Hyderabad 500034, India

**Keywords:** allo-hemodialysis, hemodialysis, kidney replacement therapy, acute kidney injury, end-stage kidney disease, green dialysis, low-resource settings, severe neonatal jaundice, dialysis adequacy, sustainability

## Abstract

Conventional hemodialysis (HD) is one of the most resource-intensive therapies in medicine and remains inaccessible to a large share of the world’s population with kidney failure. Allo-hemodialysis (alloHD) is a novel hemodialysis concept in which the blood of a healthy volunteer (“buddy”) replaces manufactured dialysate, flowing counter-currently to the patient’s blood across a conventional dialyzer membrane. Uremic solutes and excess fluid move from the patient into the buddy’s circulation and are cleared by the buddy’s own kidneys, while bicarbonate and water-soluble vitamins diffuse back to the patient. By eliminating water treatment, dialysate production, and much of a conventional machine’s electromechanical complexity, alloHD has the potential to lower the cost, complexity, and environmental footprint of kidney replacement therapy. This review synthesizes the evidence for alloHD, spanning two-compartment urea and acid–base kinetic modeling, ex vivo bench testing of a low-cost prototype, and in vivo porcine studies of acute kidney injury, while describing a modeling extension to severe neonatal jaundice. We summarize buddy safety considerations as well as review ethical issues and caregiver/health-care professional acceptance data. Finally, we position alloHD within the broader movement toward more sustainable and personalized dialysis, and outline the translational steps required before first-in-human study.

## 1. Introduction

### 1.1. An Unmet Global Need

Back in 2012, one of the authors (P.K.) encountered a woman in her late fifties, unmarried, at a hospital in Kampala, Uganda, who had begun hemodialysis (HD) about two months earlier for end-stage kidney disease (ESKD). She was receiving only one to two HD sessions per week, far below adequate dosing, when she presented with dyspnea, pitting edema, bilateral pleural effusions, and muscle wasting. Her care had been complicated, ultimately, because she ran out of funds to pay for HD treatments.

This vignette is not a rare case but rather a common example of the norm for a large portion of the world’s kidney failure population, and it frames the central question this review addresses: Can kidney replacement therapy (KRT) be re-imagined and re-engineered to be affordable enough to reach such patients?

Kidney failure remains one of the starkest examples of global health inequity. Estimates suggest that in 2010 alone, between 2.2 and 7.1 million people with kidney failure died prematurely because they lacked access to KRT [1,2]. The 2019 Global Kidney Health Atlas projected that this annual shortfall, i.e., the number of people with kidney failure who need but do not receive KRT, would grow by roughly two million between 2010 and 2030, from 7.1 to 9.1 million people [1]. More recent analyses have not narrowed this gap. After all, according to a report from the 2023 International Society of Nephrology Global Kidney Health Atlas, chronic kidney disease (CKD) is one of the ten leading causes of death globally, with over 800 million people living with this condition and roughly 3.6 million deaths attributable to kidney disease in 2021 [3]. Modeling by the Global Burden of Disease Study 2023 confirms that the global prevalence of kidney failure treated with KRT continues to rise across nearly all regions, with the steepest relative increases occurring in low- and lower-middle-income countries (LMICs), where capacity to deliver dialysis or transplantation lags furthest behind the need [4]. The 2030 projection remains sobering. Although an estimated 14.5 million people will require KRT, only 5.4 million are expected to receive it, resulting in a shortfall of roughly 9.1 million, which is consistent with the 2019 Atlas projection above, and is driven by socio-economic, infrastructural, and workforce constraints [1].

### 1.2. Conventional Hemodialysis Is Resource-Intensive

Part of the reason KRT remains unattainable for so many is structural: conventional HD, however refined, is fundamentally a complex process. A modern HD machine must produce ultrapure or at least high-quality dialysate on demand, typically 500–800 mL/min, translating into 120–192 L of dialysate for a single 4 h session and 300–800 L, once reverse-osmosis rejection rate (up to 60%) is included. A single 4 h HD session requires 7–16 kWh of electricity and produces roughly 1 kg of largely non-recyclable plastic waste [5,6,7]. The need for a continuous, chemically precise, temperature-controlled fluid stream is what necessitates the pumps, heaters, water-treatment trains, and ultrafiltration-control electronics that make a dialysis machine not only expensive (commonly USD 15,000–25,000) but also dependent on stable electricity, potable water, and trained technical staff.

In April 2025, The Kidney Disease: Improving Global Outcomes (KDIGO) group convened a Controversies Conference specifically on “Green Dialysis,” concluding that HD, hemodiafiltration (HDF), and peritoneal dialysis (PD) are among the most resource-intensive therapies in medicine, and called for new technologies and care models to meaningfully reduce this footprint [8,9]. Reducing the water and energy intensity of dialysis is therefore not a peripheral concern but a first-order clinical and public-health problem, particularly in settings where water scarcity and unreliable electricity already limit access to conventional HD.

### 1.3. Rationale for Allo-Hemodialysis

When the essential function of a HD machine is reduced to its simplest terms, it exists chiefly to prepare and safely deliver a physiologically compatible dialysate that flows counter-current to the patient’s blood, separated from it by a semi-permeable membrane. Driven by concentration and hydrostatic gradients, solutes and fluid move from the patient to the then spent hemodialysate. We reasoned that this function could, in principle, be provided by a second human being with intact kidney function, a healthy “buddy,” whose blood, rather than manufactured dialysate, flows through the dialyzer’s blood compartment counter-currently to the patient’s blood. This is the premise of allo-hemodialysis (alloHD; the Greek word állos (ἄλλος) means “other” or “another”), first presented as a concept at the American Society of Nephrology (ASN) Kidney Week in 2018 [10,11] and patented in 2019 [12]. Further developments were made through mathematical modeling, prototype engineering, ex vivo bench testing, and in vivo porcine studies [13]. Because the buddy’s blood has physiological temperature, pH, and electrolyte composition, and because the buddy’s own kidneys clear the transferred solutes and fluid, alloHD in principle eliminates the water-treatment train, dialysate production logistics, technology, and much of the sensor and control complexity of a conventional HD machine. This review summarizes the accumulated preclinical evidence for alloHD, considers the safety of the buddy role, reviews ethical analysis and early acceptance data, as well as positions alloHD within the broader effort to move HD towards more sustainable forms of KRT.

## 2. The Allo-Hemodialysis Concept

### 2.1. Basic Principle

**Figure 1:**
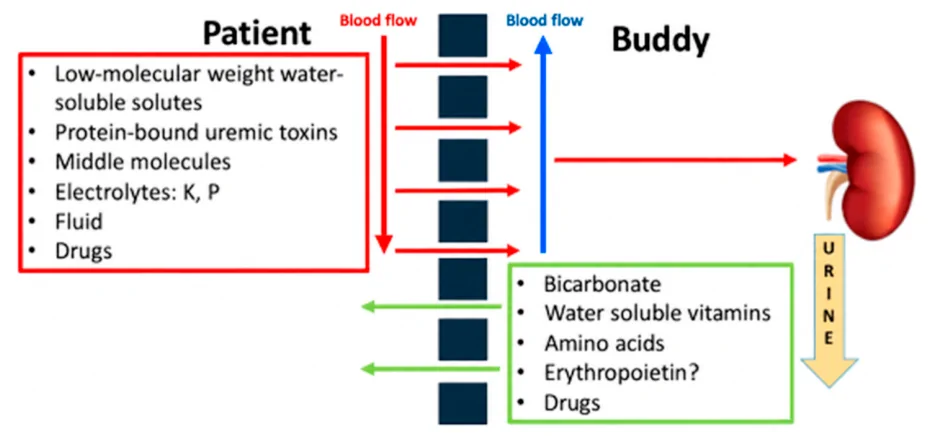
Transport of solutes and fluid across the semipermeable dialysis membrane between the patient and the buddy. The transport is driven by concentration and hydrostatic gradients. In alloHD, a healthy buddy’s blood substitutes for manufactured dialysate; the buddy’s kidneys, rather than a dialysate manufactured by a water-treatment and dialysate-production system, clear the transferred solutes and fluid. In addition, water-soluble molecules may diffuse from the buddy to the patients (e.g., bicarbonate, vitamins). The removal of protein-bound toxins is facilitated by the presence of albumin with free toxin binding sites on the buddy side.

In alloHD, the patient’s blood is drawn through a vascular access and directed into the dialysate compartment of a conventional dialyzer, while the buddy’s blood flows counter-currently through the dialyzer’s blood compartment (Figure 1). Driven by concentration and hydrostatic pressure gradients, electrolytes (e.g., potassium, phosphorus), uremic solutes (e.g., urea, creatinine, potassium, protein-bound uremic toxins) and excess fluid move from the patient into the buddy’s blood and systemic circulation. Thereafter, these solutes and fluid are cleared by the buddy’s own, functioning kidneys. The exchange is bidirectional: solutes present at relatively higher concentration on the buddy’s side, such as bicarbonate, water-soluble vitamins, and amino acids, diffuse toward the patient, contributing to correction of metabolic acidosis and, hypothetically, to nutritional replenishment, a possibility that has not been tested in vivo. In effect, the patient “borrows” renal clearance capacity from the buddy, who intermittently “lends” or “donates” it for the duration of the session [12,13]. As in conventional HD, anticoagulation of the extracorporeal circuit is required.

### 2.2. Extracorporeal Circuit and Ultrafiltration Control

An alloHD circuit needs three blood pumps, one on the patient side and two on the buddy side, whose flow-rate differential is used to generate hydrostatic pressure that drives net ultrafiltration [13]. Ultrafiltration accuracy, a safety-critical function of any dialysis machine, is achieved in the current alloHD prototype through patient and buddy extracorporeal blood flow signals from a disposable, double-channel electromagnetic flow (EMF) sensor in which the buddy’s arterial and venous blood streams pass through a single sensor housing in separate channels penetrated by a common electromagnetic field. This design cancels common-mode measurement errors and allows ultrafiltration balancing accuracy comparable to that of conventional HD machines [13]. The entire process is monitored and operated through a graphical user interface (GUI) running on a commercially available Wi-Fi-connected tablet rather than a proprietary console integrated in the alloHD machine.

### 2.3. Environmental Footprint Impact by *AlloHD* Compared to Conventional Hemodialysis

The principal comparisons and simplifications are summarized in Table 1: no water-treatment train (reverse osmosis, deionization, ultrafilters), no dialysate concentrate mixing or heating, no dialysate-side conductivity and temperature control loops, and no need for a continuous municipal water supply matched to machine-grade specifications. The lower electricity requirements for an alloHD machine may make battery or solar-supported operations more feasible than for conventional HD systems. What alloHD retains from conventional HD is the extracorporeal blood circuit itself: a dialyzer membrane, two (instead of one with conventional HD) sets of blood lines, anticoagulation, vascular access (now required in two people rather than one), and, critically, a second human being willing and medically eligible to serve as the buddy.

## 3. Preclinical and Translational Evidence

**Figure 2:**
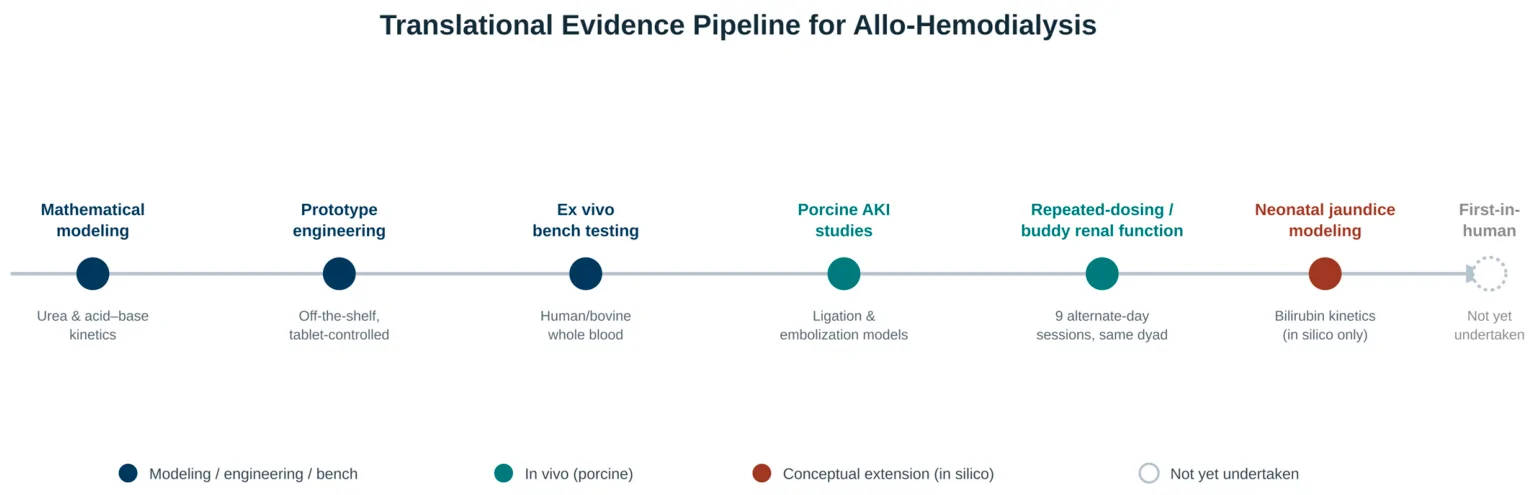
Translational evidence pipeline for allo-hemodialysis, from mathematical modeling through in vivo porcine studies to the still-preclinical neonatal jaundice modeling extension. First-in-human study has not yet been undertaken and awaits the research steps outlined in Section 8.

The evidence base for alloHD to date spans four complementary strands (Figure 2): mathematical/kinetic modeling, ex vivo bench testing of a functioning prototype, in vivo porcine proof-of-concept studies of induced acute kidney injury (AKI), and, as a conceptual extension of the alloHD principle, mathematical modeling of alloHD for severe neonatal jaundice.

### 3.1. Urea Kinetic Modeling and Dialysis Adequacy

**Figure 3:**
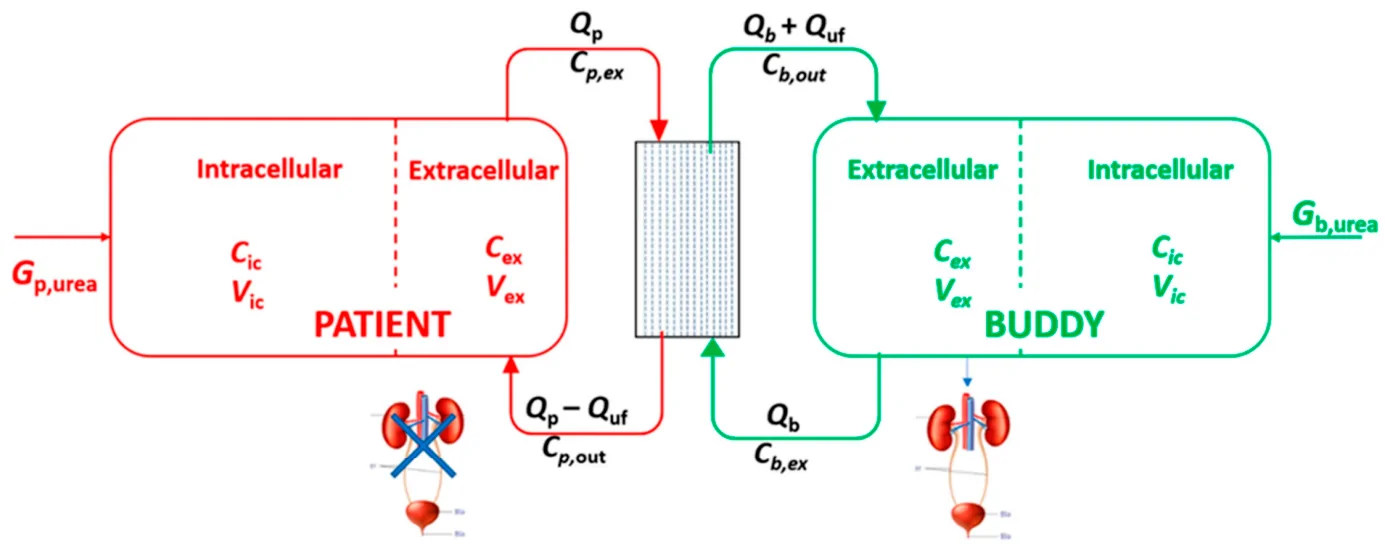
Schematic of the alloHD model, showing the patient (red) and buddy (green) connected via the dialyzer. Two-compartment (intracellular/extracellular) models are shown for both; the patient is assumed anuric, while the buddy’s glomerular filtration rate is set to 120 mL/min. Cex and Cic denote urea concentrations in the extracellular and intracellular fluid volume; Qp and Qb are the patient’s and buddy’s blood flow rates; Quf is the ultrafiltration rate; Gp,urea and Gb,urea are the urea generation rates in the patient and buddy, respectively. Reproduced from Maheshwari et al. [13] (CC BY 4.0).

A two-compartment urea kinetic model comprising intracellular and extracellular compartments was built for both the patient and the buddy, connected through a spatiotemporal dialyzer model that accounts for diffusive and convective urea transport (Figure 3) [13,14,15]. Because the buddy’s blood, unlike fresh dialysate, already contains urea, the concentration gradient driving solute removal, and hence per-session urea clearance, is inherently smaller than in conventional HD. To capture the compensatory physiology, the model also incorporated an empirical relationship linking the buddy’s urine flow rate to the transferred fluid and urea load, reflecting the fact that acute volume and solute loading of a person with intact kidneys stimulates diuresis and, secondarily, increases flow-dependent renal urea clearance [16,17].

**Figure 4:**
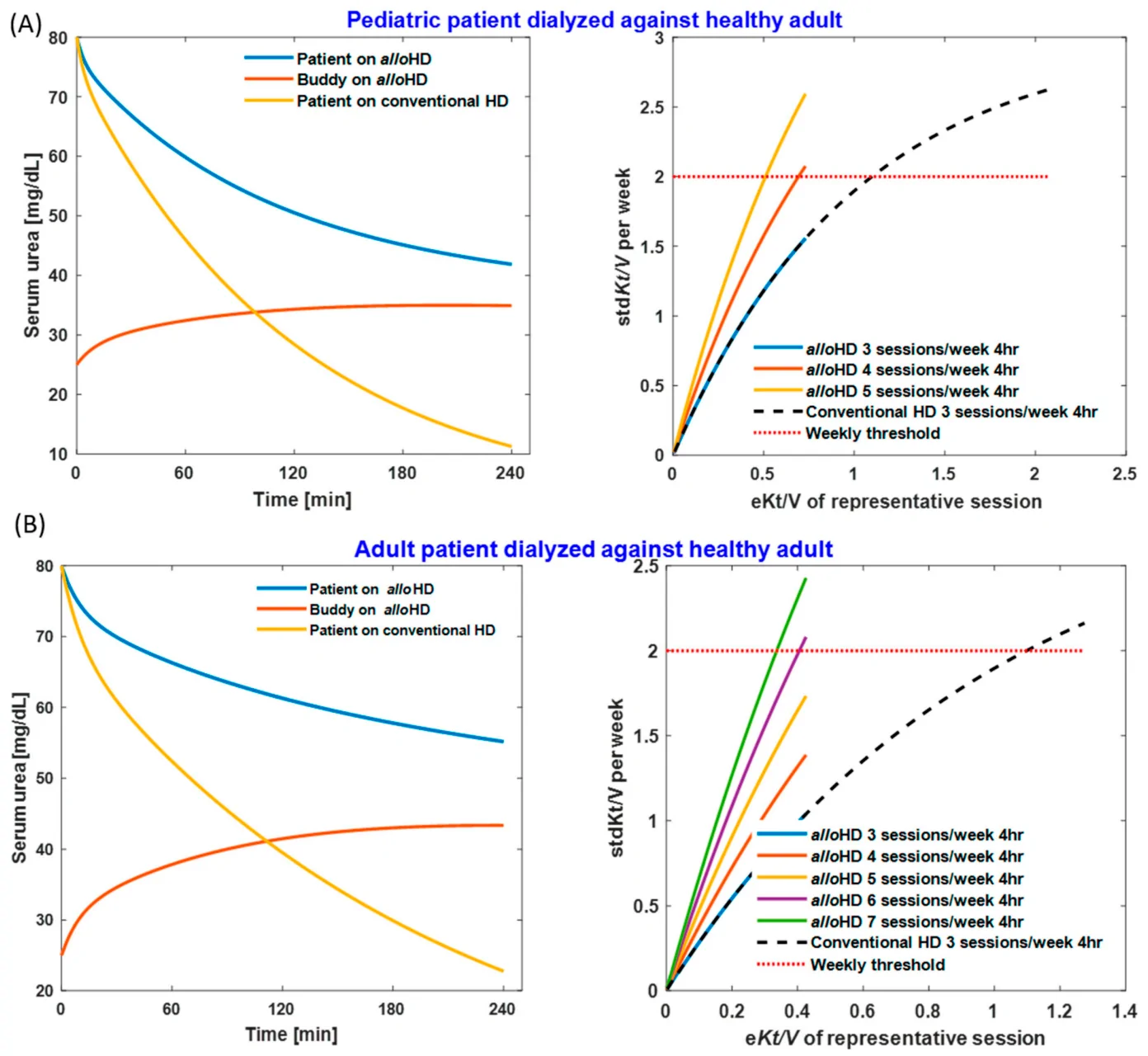
Modeled urea kinetic dynamics and dialysis adequacy. (**A**) A 20 kg pediatric patient with a patient blood flow rate of 150 mL/min, a buddy blood flow rate of 300 mL/min, an ultrafiltration volume of 1 L, and a dialyzer membrane surface area of 0.9 m^2^. (**B**) A 70 kg adult patient with patient and buddy blood flow rates of 300 mL/min each, an ultrafiltration volume of 2 L, and a dialyzer membrane area of 1.8 m^2^. In both scenarios a 70 kg healthy adult serves as the buddy. Left panels: time course of serum urea concentration with alloHD and with conventional HD (dialysate flow 500 mL/min). Right panels: standard Kt/V per week for alloHD at different weekly session frequencies versus conventional HD, referenced against the adequacy threshold recommended by the Kidney Disease Outcomes Quality Initiative (KDOQI) Clinical practice guidelines for hemodialysis adequacy, update 2006(red dotted line). Reproduced from Maheshwari et al. [13] (CC BY 4.0).

In a simulated anuric 20 kg pediatric patient dialyzed for 4 h against a 70 kg adult buddy (patient and buddy blood flow rates 150 and 300 mL/min, respectively; 1 L ultrafiltration target; 0.9 m^2^ dialyzer), alloHD achieved a urea reduction ratio (URR) of 48%, compared with 86% for conventional HD at a dialysate flow of 500 mL/min (Figure 4A). Despite this lower per-session clearance, four 4 h alloHD sessions per week were sufficient to reach a weekly standard Kt/Vurea of ≥2.0, the adequacy threshold recommended by the Kidney Disease Outcomes Quality Initiative (KDOQI) Clinical practice guidelines for hemodialysis adequacy, update 2006.

In a 70 kg anuric adult dialyzed against a 70 kg buddy (blood flow rate 300 mL/min on both sides, 2 L ultrafiltration target, 1.8 m^2^ dialyzer), alloHD achieved a URR of 31% versus 72% for conventional HD, and six 4 h sessions per week were required to reach the same weekly adequacy threshold (Figure 4B) [13]. Mechanistically, the model shows that the buddy’s serum urea concentration does not rise unbounded over a session but plateaus, because the transferred urea and fluid load progressively increase the buddy’s urine output and urea clearance. In the pediatric–adult scenario, the buddy’s urine volume increased by an estimated 182 mL over 4 h (18% of the 1 L ultrafiltration volume), and in the adult–adult scenario by 558 mL (28% of the 2 L ultrafiltration volume). Detailed modeling considering both fluid volume and transferred urea show that the buddy’s urine volume would normalize to pre-alloHD levels after about 24 h (see Supplemental Material in [13]).

### 3.2. Acid–Base Correction

**Figure 5:**
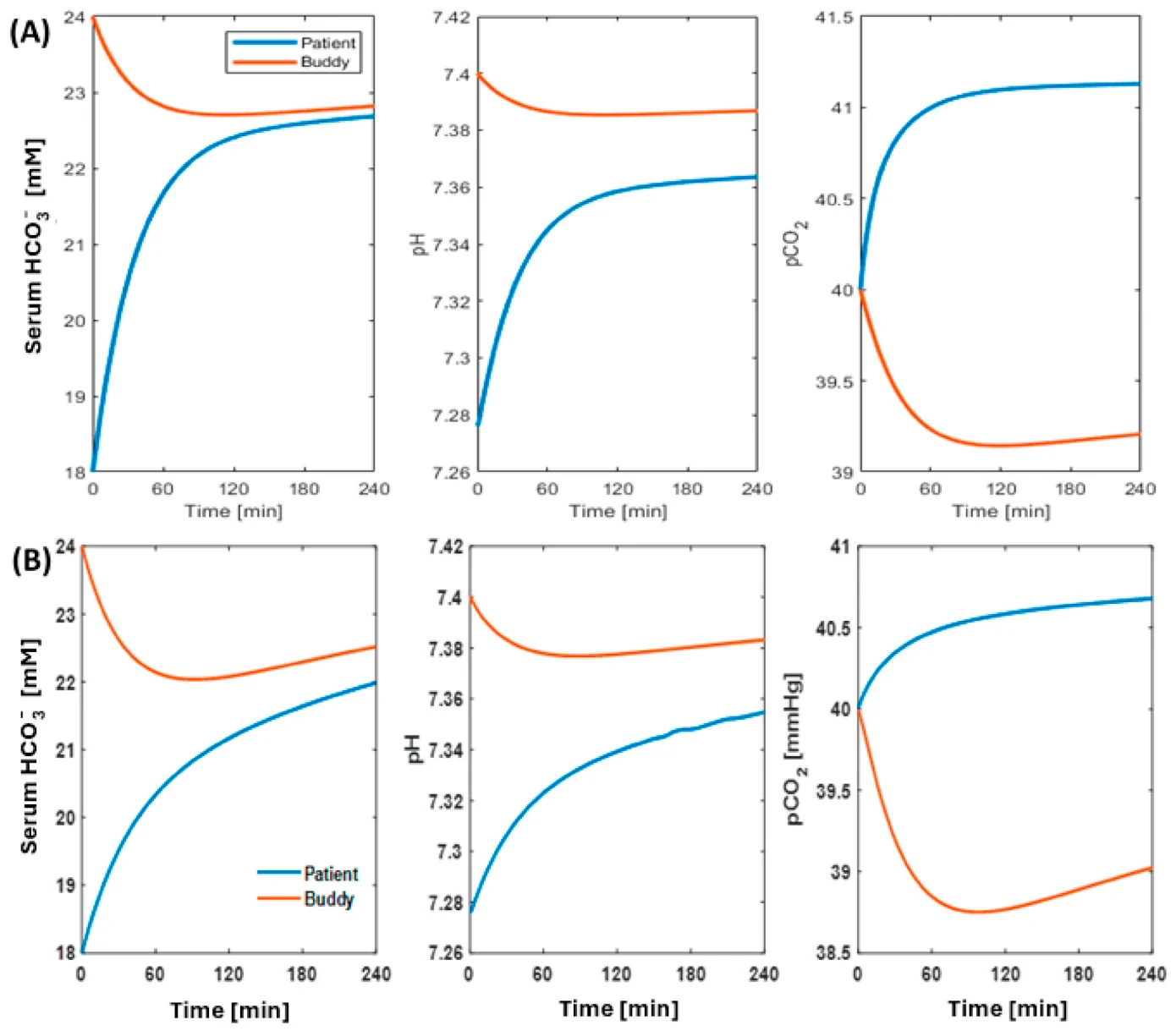
Modeled temporal dynamics of acid–base status in the patient (blue) and buddy (red) during alloHD. Panel (**A**) shows the pediatric patient–adult buddy dyad; panel (**B**) shows the adult–adult dyad. Serum HCO_3_^−^, pH, and pCO_2_ are shown over a 4 h alloHD session. The buddy’s acid–base status shows only minimal, self-limited perturbation, consistent with intact renal regulation. Reproduced from Maheshwari et al. [13] (CC BY 4.0).

Using a previously published physiological model of the bicarbonate/CO_2_ buffering system [18], configured to couple patient and buddy compartments via the alloHD circuit, simulations showed that a single 4 h alloHD session can meaningfully correct metabolic acidosis. Starting from a pre-dialysis serum bicarbonate of 18 mmol/L in the (anuric) patient, the adult–adult simulation predicted a rise to approximately 22 mmol/L, with pH increasing to roughly 7.36 and only a minimal rise in pCO_2_ (from 40 to ~40.7 mmHg); the pediatric–adult scenario showed a similar, slightly more favorable trajectory. Notably, the buddy’s own acid–base status, protected by intact renal regulation, showed only a modest and self-limited perturbation, with HCO_3_^−^, pCO_2_, and pH dipping over the first two hours before the buddy’s own renal and respiratory compensation restored baseline values (Figure 5).

### 3.3. Protein-Bound Uremic Toxin Transport

Because the dialyzer membrane and transport physics of alloHD are otherwise identical to conventional HD, prior modeling work on protein-bound uremic toxin (PBUT) kinetics in HD and HDF is directly applicable to alloHD’s dialyzer-level solute transport and provides a mechanistic basis for the ex vivo PBUT removal [14,15]. A plausible [19] advantage of alloHD for PBUT clearance is that the buddy’s blood could provide a continuous supply of albumin with unoccupied PBUT-binding sites on the “dialysate” side of the membrane, which might sustain the diffusive gradient for protein-bound solutes for longer than an albumin-free conventional dialysate stream would and thus enhance PBUT clearance. While untested in vivo, ex vivo experiments using whole blood, with the patient bucket initially spiked with indoxyl sulfate and p-cresyl sulfate, support that concept, because the solute concentration differences between the buddy and patient buckets dissipated rapidly within 15–20 min [13], much faster than observed with conventional HD.

### 3.4. Prototype Engineering and Ex Vivo Bench Testing

**Figure 6:**
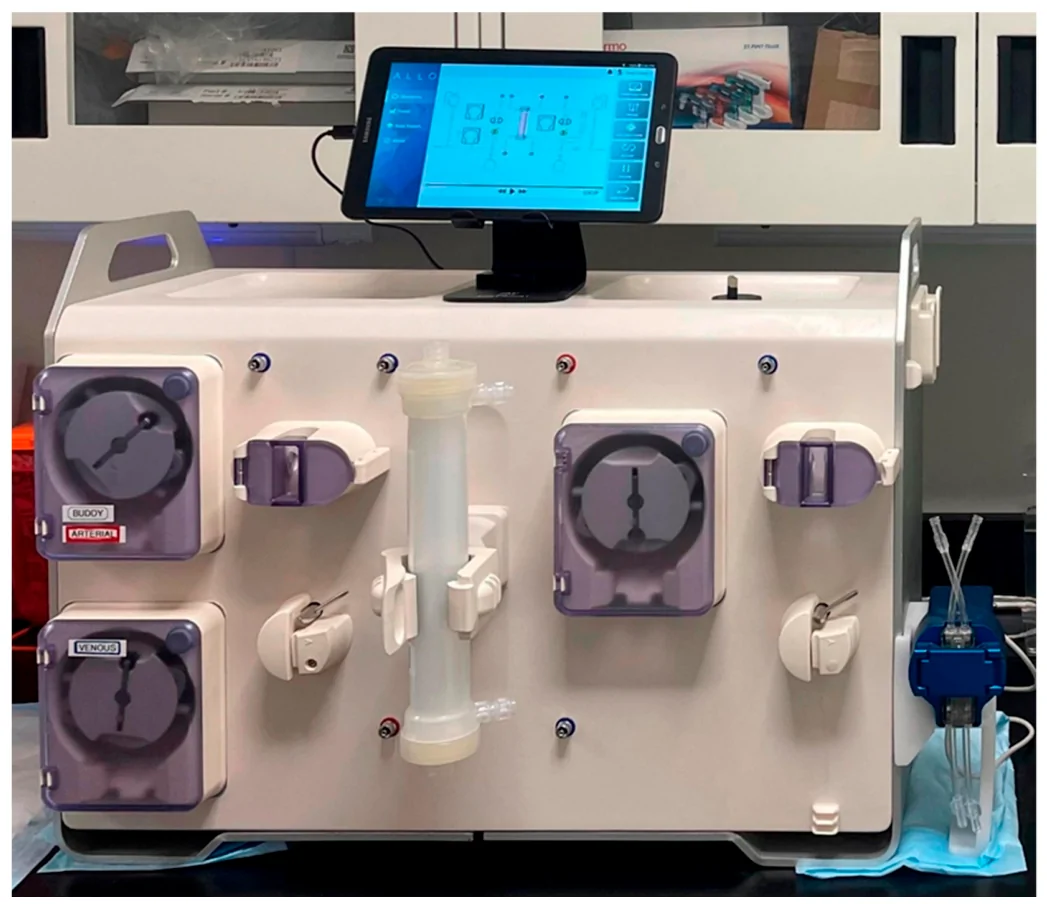
The alloHD machine prototype. Three peristaltic blood pumps are visible, two on the buddy side (**left**) and one on the patient side (**right**), along with the dialyzer and electromagnetic flow balancing sensor. The device is connected via Wi-Fi to an Android tablet running the graphical user interface (shown on top). Reproduced from Maheshwari et al. [13] (CC BY 4.0).

To move beyond simulation, an alloHD machine prototype was designed and built in under two months using exclusively off-the-shelf hardware, controlled through a commercially available Android tablet rather than a proprietary console. The completed device weighs approximately 27.6 kg and measures roughly 64 × 32 × 47 cm, with firmware distributed across a real-time microcontroller (pump control, ultrafiltration control, pressure and venous-clamp monitoring) and a Linux-based co-control board (Wi-Fi communication, balancing-sensor control), all exposed through an Android GUI for system configuration, status display, and data logging (Figure 6) [13].

Ex vivo bench experiments with human whole blood (500 mL patient and buddy buckets, 1.7 m^2^ high-flux cellulose triacetate dialyzer, ~110 mL/min blood flow on both sides) showed that spiked urea and potassium equilibrated between the patient and buddy compartments within 30–45 min, with the estimated urea mass transfer coefficient (KoA, 853 mL/min) closely matching model predictions. In a separate experiment, creatinine equilibrated within 5 min, while the PBUTs indoxyl sulfate and p-cresyl sulfate equilibrated more slowly (15–25 min), consistent with their protein binding [13]. Across all ex vivo runs, no blood clots were observed even after 2 h of recirculation, and no hemolysis was detected. In a dedicated ultrafiltration-performance test using 4 L of bovine blood on each side of the dialyzer over a 3 h session, the prototype’s control system achieved a 750 mL ultrafiltration target in a smooth, linear fashion, corroborated independently by a gradual decline in patient-bucket weight and in buddy-blood hematocrit [13].

### 3.5. In Vivo Porcine Studies

Building on the ex vivo bench-test results, alloHD has been evaluated in a series of porcine studies reported at several international conferences. These porcine studies were conducted in Hyderabad, India under approval of the Committee for the Purpose of Control and Supervision of Experiments on Animals (CPCSEA). Female Yorkshire pigs with central-venous vascular access were dialyzed against one another (rather than against an AKI model), for 2–4 h, one to three times weekly, using a Nipro Cellentia 17H dialyzer, systemic heparin anticoagulation (5000 IU/h), and ultrafiltration volume goals of 0–1000 mL. Across 10 completed alloHD sessions, visual inspection showed no evidence of hemolysis, a finding corroborated in seven of these experiments by plasma lactate dehydrogenase and haptoglobin measurements in both “patient” and buddy pigs; no dialysate-compartment coagulation was observed [20].

AlloHD was then evaluated in a porcine AKI model. Four female Yorkshire pigs (30–80 kg) received central-venous vascular access; AKI was induced in two animals by surgical ligation of renal blood vessels, and each AKI pig was dialyzed for 4 h against a separate healthy buddy pig (one alloHD session per pair), again using a Nipro Cellentia 17H dialyzer and heparin anticoagulation. Blood urea nitrogen (BUN) and creatinine declined in both AKI animals over the session while rising transiently, then declining, in the corresponding buddy animals during the second half of dialysis; no hemolysis or dialyzer coagulation was observed [21].

**Figure 7:**
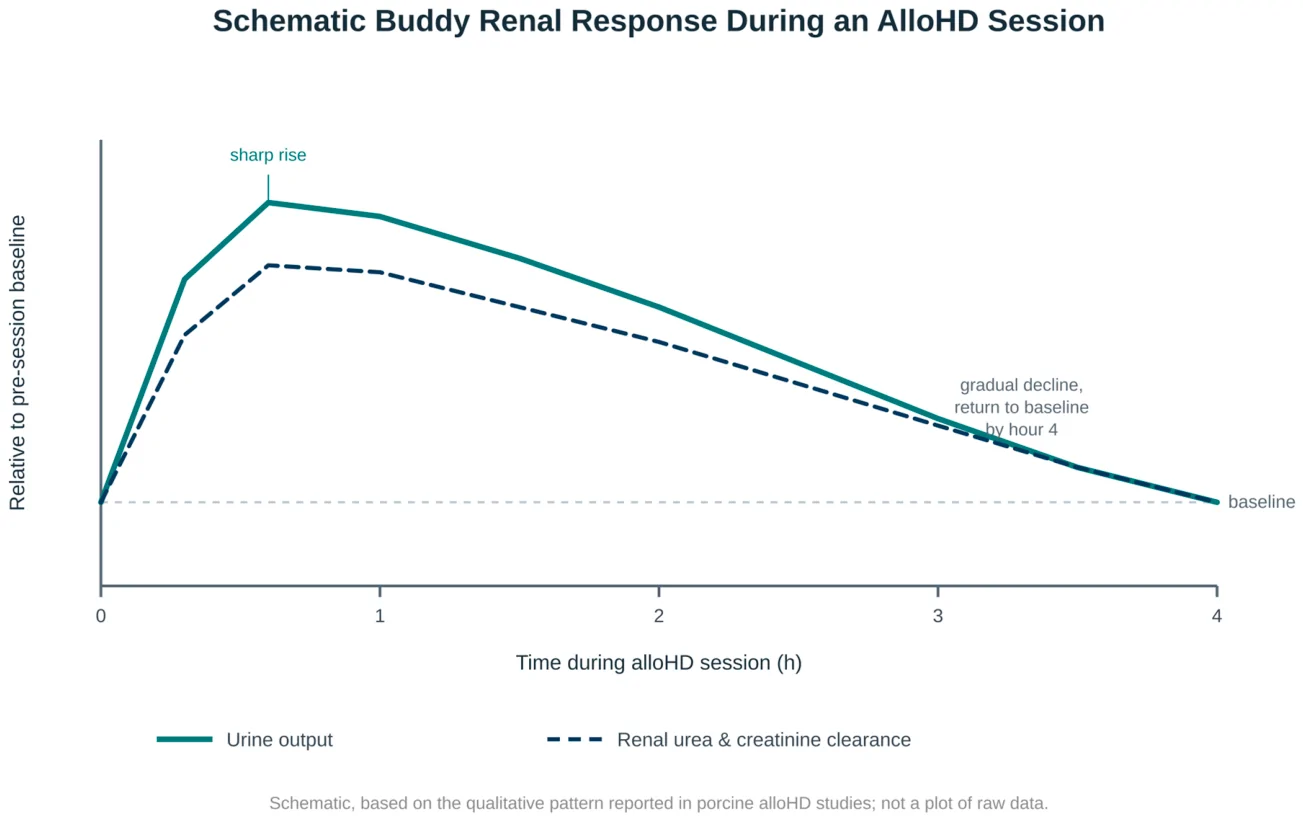
Schematic representation of the buddy’s renal response during a single alloHD session, based on the qualitative pattern reported in repeated-dosing porcine studies: urine output rises sharply at session initiation, declines gradually over the following hours, and returns to pre-session baseline by the end of a 4 h session, with renal urea and creatinine clearance following a parallel trajectory. This panel is illustrative of the reported pattern rather than a plot of the underlying raw data [23].

A subsequent study used a less invasive, catheter-based model of AKI—bilateral transcatheter renal-artery embolization with polyvinyl alcohol particles—in two female Yorkshire pigs (80–90 kg), again connected via central-venous access to the dialysate (AKI pig) and blood (buddy pig) compartments of a Nipro Cellentia 17H dialyzer. Nine alternate-day, 4 h alloHD sessions were performed with this single AKI–buddy pair (i.e., repeated exposure of one dyad rather than replication across multiple animal pairs); plasma BUN and creatinine declined in the AKI pig during each session while rising transiently above baseline in the buddy pig before trending back toward baseline post-dialysis, a pattern that was consistent across all nine sessions (Figure 7). No hemolysis or coagulation of the extracorporeal circuit was observed in any session, and the animals were sacrificed after the ninth session [22].

Building on this dataset, further analysis specifically examined the buddy’s own kidney function across the same nine sessions, adding hourly urine collection from the buddy pig via an indwelling urinary catheter and calculation of urea and creatinine clearance. Buddy urine output rose sharply after alloHD initiation, decreased gradually over the first three hours, and returned to normal during the final (fourth) hour of each session; buddy renal urea and creatinine clearances (normalized to body weight) showed a parallel initial rise followed by a gradual return toward baseline, remaining within or close to normal porcine reference ranges (urea clearance 9–18 mL/min/10 kg body weight; creatinine clearance 15–34 mL/min/10 kg) throughout (Figure 7). No deterioration was detected in the measured short-term functional parameters across the nine repeated sessions [23].

Taken together, these in vivo results corroborate the central mechanistic premise identified by the mathematical model, namely, that transient fluid and solute loading of a healthy kidney pair, repeated on an alternate-day schedule, was accompanied by compensatory diuresis and clearance responses in one repeatedly studied dyad. As with the embolization study above, these findings derive from a single dyad studied repeatedly rather than from independent replication across multiple animal pairs.

### 3.6. A Conceptual Extension: Allo-Hemodialysis for Severe Neonatal Jaundice

The same buddy-as-dialysate principle has been explored, so far only through mathematical modeling, as a potential treatment for severe neonatal jaundice (SNJ). SNJ is a condition with a disproportionate burden in LMICs, where an estimated 481,000 term or near-term infants are affected annually, with roughly 114,000 deaths and 63,000–75,000 survivors developing kernicterus spectrum disorder (cerebral palsy, sensorineural hearing loss, and other neurodevelopmental sequelae) [24]. Term/near-term neonates with severe unconjugated hyperbilirubinemia (>30 mg/dL) and low-albumin (<3.0 g/dL) and high-neurotoxicity-risk infants requiring escalation to exchange transfusion are considered the targeted patient population.

In this application, the neonate’s blood would flow through the hollow fibers of a miniaturized (0.075 m^2^) dialyzer at 12.5–15 mL/min (for a 2.5–3.5 kg infant), counter-current to an adult buddy’s blood at 30 mL/min through the dialysate compartment. Modeling unconjugated bilirubin kinetics under physiologically plausible albumin levels (1.1–3.1 g/dL in the neonate, 3.3 g/dL in the buddy) suggested that a single 6 h allo-HD session could lower neonatal bilirubin by more than 35%, with greater relative benefit at lower neonatal albumin levels, precisely the setting in which unbound, neurotoxic bilirubin risk is highest. Because of the adult buddy’s much larger bilirubin distribution volume and intact hepatic conjugation capacity, the buddy’s own bilirubin level was predicted to rise only transiently [25]. If corroborated by ex vivo, animal, and eventually clinical studies, this application could offer a potentially lower-cost, extracorporeal complement or alternative to exchange transfusion or phototherapy in settings where these interventions are unavailable or high-risk. At this stage it remains a modeling result requiring substantial further validation before any clinical inference can be drawn. Regarding the buddy, we would anticipate that parents and/or relatives of the newborn would likely take on this role.

## 4. Health and Safety Considerations for the Buddy

Because alloHD is, by design, the only dialysis modality in which a second, healthy person’s physiology is directly incorporated into the treatment circuit, buddy safety is the central and most distinctive risk domain requiring evaluation. Regarding buddy eligibility, key medical criteria should be assessed before acceptance, including normal kidney function and urinalysis; absence of HIV, hepatitis B, and hepatitis C infection; and either normal blood pressure or well-controlled hypertension. Potential psychosocial criteria could be based on the model by Ashkenazi et al. [26] and may include the absence of mood disorders, and ability to miss work or school plus the ability to understand their risks (e.g., vascular access, infection) as they sign an informed consent.

### 4.1. Infection and Cross-Contamination Risk

The dialyzer membrane is, in principle, a robust barrier against pathogen transfer: the pore diameters of contemporary low-flux hollow-fiber membranes range from roughly 1.1 to 3.5 nm, well below the size of the smallest viruses of clinical concern (parvovirus B19, 18–26 nm; hepatitis B virus, ~42 nm; hepatitis C virus, 55–65 nm; HIV, 100–160 nm) [13,27]. Based on this size relationship, transfer of intact virions across an intact membrane is considered highly unlikely. The more realistic risk is membrane rupture or blood-line leak leading to direct cross-contamination of patient and buddy blood; FDA MAUDE adverse-event data from 2016 to 2019 suggest such leaks occur at a rate of roughly two to three per million conventional HD sessions [13]. Because alloHD, unlike conventional HD, places two people’s blood in direct proximity across a single membrane, pre-treatment screening of both patient and buddy for transmissible infections (HIV, hepatitis B/C, and other regionally relevant pathogens) is regarded as an essential safeguard, and reliable, real-time blood-leak detection remains an open engineering requirement specific to alloHD.

### 4.2. Hemocompatibility of Dialysate Compartment Blood Flow

Flowing blood through what is normally the dialysate compartment of the dialyzer has, in prior hemofiltration research, been associated with a reduced, rather than increased, propensity for clot formation [28]. This is consistent with the absence of clinically significant coagulation observed across the ex vivo bench experiments (up to 2 h of recirculation) and the in vivo porcine sessions (up to 4 h, repeated over nine alternate-day treatments) reviewed above. Since systemic anticoagulation (heparin) was used throughout these studies, buddy-side dosing and monitoring will require dedicated evaluation in any human protocol.

### 4.3. Buddy Renal and Systemic Adaptation to Intermittent Solute and Fluid Loading

The physiological plausibility of the buddy role is supported by the porcine data summarized in Section 3.5 and by the independent living-kidney-donation literature, which documents human tolerance of acute reductions in effective nephron mass [29]. Compared with donor nephrectomy, alloHD imposes a time-limited solute and fluid load without loss of nephron mass. The magnitude and reversibility of this physiological challenge will depend on patient–buddy size and capacity matching, treatment intensity, and repeated exposure, and have not yet been established in humans; the porcine finding that buddy renal clearance and urine output return toward baseline within the same session and show no apparent cumulative decline over nine repeated exposures in a single dyad [23] is reassuring but preliminary. The living-donor analogy is, likewise, reassuring but indirect. It demonstrates renal reserve after permanent nephron loss, not the safety of recurrent acute solute and volume loading combined with vascular access and extracorporeal circulation, a different physiological exposure whose own risks (anticoagulation, repeated cannulation) are not necessarily reversible. Living donors are also extensively pre-screened for renal reserve and undergo the challenge only once, whereas a chronic alloHD buddy would be exposed repeatedly; the long-term cumulative effect of such repeated exposure in humans has not yet been studied and constitutes one of the more important open questions discussed in Section 8.

A related, and only partly resolved, question concerns buddy fluid balance. In the modeled adult–adult scenario, the patient’s 2 L ultrafiltration target is offset by only an estimated 558 mL of additional buddy urine output over the same 4 h session [13], leaving roughly 1.4 L of transferred fluid within the buddy’s extracellular compartment at session’s end (a smaller, analogous gap, 182 mL against 1 L, occurs in the pediatric–adult scenario). Extended modeling suggests this residual volume is excreted within 24 h (Supplemental Material in [13]), but this has not yet been confirmed in humans or animals. This is likely to be well tolerated by a healthy adult buddy after a single session, but it becomes a more important question for smaller buddies, larger ultrafiltration prescriptions, or repeated chronic exposure. The question is a concrete, quantifiable gap for future modeling and animal studies to address.

### 4.4. Patient–Buddy Physiological Matching and Capacity Limits

The modeling and porcine data reviewed above are most reassuring for a specific, favorable configuration: a small, anuric patient dialyzed against a much larger, healthy adult buddy. The 20 kg pediatric patient–70 kg adult buddy dyad (Section 3.1) illustrates this well, and it is not a coincidence that this is also the configuration in which urea kinetic modeling looks most favorable and buddy urine and clearance responses are proportionally smallest relative to buddy renal reserve. The relationship is far less established for less favorable dyads—for example, a large or highly catabolic adult patient paired with a considerably smaller buddy. Both the efficacy and the safety of alloHD are therefore likely to depend on a formal, prescription-specific relationship between the patient’s solute and fluid burden and the buddy’s size and physiological reserve, a relationship we term patient–buddy physiological matching.

Relevant factors of physiological matching likely include the buddy’s glomerular filtration rate; the relationship between transferred fluid volume and the buddy’s own extracellular volume and total body water; the patient’s solute burden and ongoing generation rates; the buddy’s renal handling of potassium, phosphate, acid, and water; the buddy cardiovascular reserve and tolerance of acute volume loading; extra-renal metabolic capacity, since not all transferred solutes are cleared renally; the prescribed ultrafiltration volume relative to buddy body size; treatment duration and frequency; and the recovery interval between exposures. Urea is probably among the least physiologically demanding of the transferred solutes; potassium, phosphate, acid load, middle molecules, protein-bound uremic toxins, and medications may present a greater challenge to buddy homeostasis, and several of these depend substantially on extra-renal rather than renal handling.

Two practical implications follow. First, buddy eligibility screening for alloHD cannot be reduced to an assessment of normal versus abnormal kidney function; it must instead weigh a candidate buddy’s specific physiological reserve against a specific patient’s prescription. Unfavorable patient–buddy combinations may require a modified prescription (e.g., reduced ultrafiltration rate, shorter sessions, a larger or otherwise more suitable buddy) or may not be appropriate for alloHD at all. Second, future modeling should deliberately explore unfavorable dyads, and future animal and eventually human studies should measure dynamics of electrolyte, acid–base, volume, and biomarker responses in the buddy, together with the time course of recovery.

This consideration is relevant to the choice of an initial clinical indication (Section 7): the favorable patient–buddy size relationship in pediatric AKI may be central to the safety and feasibility of alloHD as currently understood. The first human studies of alloHD should likely be confined to the most favorable physiological configuration, a small patient and a carefully screened, appropriately larger adult buddy with modest treatment requirements, before any extension to less favorable, and currently less well-characterized, patient–buddy combinations. Pediatric AKI may represent a physiologically favorable eventual indication because of patient–buddy size matching and finite treatment duration. Whether it is appropriate as the first-in-human indication will require independent ethical and regulatory assessment and sufficient prior safety evidence: children are a vulnerable research population, and the Declaration of Helsinki requires that their inclusion be justified by the inability to first answer the relevant questions in a less vulnerable group.

The evidence supporting alloHD remains preliminary and heterogeneous. Mathematical models and laboratory experiments address selected aspects of the concept but cannot establish clinical safety or effectiveness. Comparisons with established medical procedures may inform risk assessment and protocol development, but differences in exposure, physiology, and participant characteristics limit their applicability. In particular, these analogies cannot establish the safety of repeated alloHD exposure for patients or buddies. Clinical feasibility and safety therefore remain open questions requiring clinical studies.

### 4.5. Vascular Access

Achieving blood flow rates adequate for meaningful clearance (150–300 mL/min in the modeling and bench-test scenarios reviewed above) will likely require central-venous or arteriovenous vascular access in the buddy as well as the patient for any chronic treatment program. This poses a nontrivial additional procedural burden and risk for the buddy. Precedent for creating vascular access in individuals without kidney failure exists in other clinical contexts, including arteriovenous fistulae used for automated red-cell exchange in sickle-cell disease, for long-term venous access in hemophilia, and for home parenteral nutrition, where reported experience has generally been favorable [30,31,32]. Whether similar access strategies would be acceptable, and durable, for an intermittent, non-therapeutic (to the buddy) purpose such as alloHD is an open question requiring dedicated study.

## 5. Ethical and Social Dimensions

### 5.1. Early Ethical Consultation

Rather than treating ethical review as a late-stage regulatory hurdle, the investigators deliberately built independent ethical consultation into the earliest phase of the project, engaging a medical ethicist immediately after mathematical modeling had initially demonstrated the theoretical viability of alloHD, and well before any prototype was built. This consultation began with Professor Nicholas Steneck (Professor Emeritus of History of Science, University of Michigan; independent research integrity consultant; former Director of the Michigan CTSA Ethics Program), who was posed two framing questions.

1. Are there compelling ethical reasons against further research into alloHD? Steneck’s assessment was that alloHD does raise legitimate ethical questions, but that none of them argues against continued research; given the scale of the problem, millions of people without access to affordable KRT, there is an affirmative ethical mandate to explore alternative treatment options, and given that access to treatment depends heavily on economic status, an equally strong mandate to explore options, such as alloHD, capable of serving low-resource settings.
2. If alloHD offers an effective option relative to conventional KRT, can it be used ethically? Here the assessment was conditional: this could plausibly be so, but final judgment would depend on the outcome of research findings, with the most significant open issue being risk to the patient and, especially, the buddy. If alloHD were found to increase risk relative to conventional therapy, equitable distribution of that risk and questions of buddy compensation would need to be addressed; conversely, it was noted that alloHD might instead decrease patient risk, create legitimate employment for the buddy, lower overall health-care costs, or produce other unanticipated benefits.

Further consultation raised no clear ethical concerns, pending further information on safety.

With safety being raised as the key substantive issue requiring resolution before human study, a staged approach to addressing this issue was adopted, progressing first to animal studies. If animal studies raised no major safety concerns, the appropriate next step would be to design and submit protocols for clinical trials in living patients and buddies to the relevant institutional review board(s). If animal studies support progression but leave specific, limited safety questions unresolved, a decedent alloHD study could provide an intermediate step before studies in living participants. By connecting two decedents maintained on circulatory support, this approach could permit assessment of solute clearance and acute immune and complement responses in a human physiological setting, although it would not establish long-term safety. Then, once sufficient animal research had established reasonable confidence in safety, appropriately designed, IRB-approved clinical trials would be the next legitimate step in the evaluation of alloHD, with the understanding that final decisions on ethics rest with the procedure and individuals responsible for the protection of human subjects in research.

### 5.2. Caregiver and Health-Care Professional Acceptance

Because alloHD, uniquely among dialysis modalities, requires the recurring participation of a second person, its acceptability to potential buddies and to the clinicians who would prescribe it is a first-order feasibility question, distinct from its technical performance. In a cross-sectional survey conducted in Morelia, Mexico, Campos and colleagues asked care partners related (n = 33) and unrelated (n = 42) to patients with end-stage kidney disease, as well as health-care professionals (n = 50, predominantly physicians), whether they would be willing to serve as an alloHD buddy for the person they cared for [33]. A majority responded affirmatively, 76% of related caregivers, 67% of unrelated caregivers, and 44% of health-care professionals. Even larger majorities agreed with the alloHD procedure in principle (88%, 91%, and 64%, respectively). Notably, willingness to donate kidney function intermittently via alloHD was somewhat lower than stated willingness to donate a kidney outright (95%, 91%, and 98% in the same three groups). One reason for that response could have been that in the ESKD scenario kidney donation is potentially a cure while alloHD is not. These findings should be interpreted within Mexico’s cultural, religious, familial, and health-care context and may not be generalizable to other populations or health-care systems. Also, the response may look different when asked about a pediatric AKI scenario.

This comparison, however, may understate true willingness for alloHD’s most plausible early applications. Serving as an alloHD buddy for a patient with self-limited AKI, or for a neonate with severe jaundice (Section 3.6), implies a finite commitment of days to at most a few weeks, fundamentally different from the lifelong physiological commitment of kidney donation; the Campos survey asked about willingness in general or explicitly chronic terms rather than distinguishing these scenarios. Stated willingness would plausibly be higher when the comparison is framed around a defined, time-limited course of treatment for a recoverable condition rather than an open-ended or lifelong commitment. Refining acceptance research along these lines, explicitly separating temporary, AKI-, and neonate-specific indications from chronic maintenance dialysis, is an important next step, and is consistent with this review’s broader conclusion that pediatric and neonatal AKI, rather than chronic ESKD, may represent alloHD’s most physiologically favorable eventual indications, pending the independent ethical and regulatory assessment discussed in Section 4.4.

Consistent with this survey signal, informal discussion of alloHD with approximately 100 physicians across more than 20 countries and six continents has suggested general agreement that the concept is technically plausible. The vast majority of consulted physicians indicated that pediatric AKI may represent the most promising near-term clinical application, with reliable vascular access solutions for the buddy remaining as the most significant translational barrier.

### 5.3. Family/Caregiver Impact of alloHD

Several publications have described the impact of KRT on the family/caregiver [34,35]. The areas described include caregiver time and cost strain, social/life challenges and sleep deprivation. A PRISMA systematic review of 16 manuscripts revealed that caregivers experience higher burden, anxiety, depression, and lower quality of life and overall well-being [36]. The impact of alloHD on caregiver physical/mental health, quality of life and well-being, will need to be investigated. Strategies to reduce caregiver burden may need to include the five areas identified by Nurse Fatona’s literature review [37] such as self-management model, stress management training, online peer mentoring programs, the teach-back method, and peer support intervention.

### 5.4. Equity, Coercion, and Compensation

If alloHD was shown to be safe and effective, questions of equitable risk distribution, buddy compensation, and protection against coercion would require careful, context-specific attention. This is particularly important in LMIC where socioeconomic pressure could distort ostensibly voluntary participation, echoing long-standing debates in living organ and tissue donation ethics. An additional aspect is dual standard of care, where a less efficient therapy is offered to the poor vs. advanced therapy to the affluent. However, for many patients with kidney failure in LMIC, the direct comparator is not advanced KRT, but death, as for many of target population there may be no kidney replacement therapy at all. Proponents have noted that alloHD could plausibly create a legitimate, regulated source of income for buddies, analogous to compensated plasma or blood donation models in some jurisdictions and might lower overall treatment costs relative to conventional in-center HD. However, neither the health-economic case nor an appropriate compensation and consent framework has yet been formally studied.

### 5.5. The Ethics of Prioritization: Is It Ethical Not to Pursue AlloHD?

The ethical consultation described in Section 5.1 was framed, at the outset of this research program, around a single question: Are there compelling ethical reasons against further research into alloHD? Given the feasibility evidence assembled in this review, we think the more pressing question has shifted. Millions of people with kidney failure continue to die prematurely each year for lack of affordable treatment (Section 1.1), and neither of the two most commonly proposed long-term solutions is likely to close this gap for the population currently affected. Incremental improvements that make conventional dialysis modalities technology cheaper, greener, or more efficient remain worth pursuing but are, by their nature, gradual. Xenotransplantation or other manufactured-kidney approaches, if realized at scale, would address today’s organ-supply constraint but not necessarily the cost constraint: a transplantable kidney, however produced, seems unlikely to be affordable to the LMIC populations who bear the largest share of the current treatment gap. AlloHD, by contrast, is a low-cost, low-complexity approach with a plausible mechanism and an accumulating, if still preclinical, feasibility record, whose remaining barriers are translational and regulatory rather than fundamental.

This asymmetry raises a question distinct from, though related to, the one addressed in Section 5.1: given an approach capable of providing at least an interim solution to a problem currently costing millions of lives, what obligation exists to prioritize its further development, in research funding, institutional support, and regulatory attention, alongside, rather than after, longer-horizon efforts to reform conventional dialysis? We do not think this review can answer that question on its own and are conscious of the risk of overstating evidence that remains preclinical. But we think the burden of justification should run in both directions. Those who prioritize incremental improvement of established technology over pursuit of alternative approaches such as alloHD should be prepared to explain why patients priced out of care today should wait for solutions that, however promising, offer no comparable near-term prospect of reaching them.

Current evidence for alloHD is preliminary and does not establish its clinical feasibility, safety, or affordability. Although the approach may reduce equipment costs, its overall economic implications will depend on staffing, consumables, screening, monitoring, and local health-care conditions and require formal evaluation. Similarly, unmet need for kidney replacement therapy motivates further investigation but does not establish the ethical priority of alloHD over alternative strategies. Future assessments must consider patient benefit, buddy safety and burden, acceptability, and the opportunity costs of implementation.

## 6. Allo-Hemodialysis Within the Broader Effort to Redefine Hemodialysis

This Special Issue’s central premise that HD must move beyond one-size-fits-all delivery toward more precise, physiology-aware, and outcome-oriented therapy is usually approached from the technology side, e.g., higher-convective-volume hemodiafiltration, next-generation membranes with tailored sieving properties, and AI-enabled prescriptions that are continuously adaptive [38]. AlloHD approaches the same underlying problem, namely that conventional HD imperfectly replaces native kidney function while consuming disproportionate resources to do so, from a different direction. Rather than engineering an increasingly sophisticated machine to approximate physiology, alloHD builds on existing physiology itself, in the form of a healthy buddy’s blood and kidneys. In terms of transport-physics, alloHD remains fundamentally diffusive and convective; its innovation lies not in the mass-transfer mechanism but in the source of the counter-current fluid and in the potential reduction in water-treatment and dialysate-production requirements this affords.

This positions alloHD as a complementary rather than competing innovation within the “redefining hemodialysis” agenda. Whereas advanced membranes, high-volume HDF, and AI-guided prescriptions aim to attain more precise, personalized clearance from patients who already have reliable access to sophisticated dialysis infrastructure, alloHD targets the much larger population for whom that infrastructure (the water, electricity, and technical workforce it requires) remains unavailable [1,2,3] and in austere environments [39]. In this sense alloHD is a “precision” therapy in a different register: precision not in molecular-weight-selective clearance, but in matching the level of technology deployed to the resource environment in which it must operate, while retaining the option, in principle, of applying the same membrane and mass-transfer advances discussed elsewhere in this issue to the alloHD circuit itself as the concept matures. It also aligns directly with the “green dialysis” strand of this agenda by eliminating dialysate production altogether rather than incrementally reducing dialysate flow or recycling reverse-osmosis reject water. We propose that alloHD represents a structurally different, rather than merely more efficient, approach to the water and energy burden that the recent KDIGO Controversies Conference identified as a first-order challenge for the field [8].

## 7. Potential Candidate Clinical Use Scenarios

The primary aim of alloHD is to expand access to affordable, potentially lifesaving treatment in LMICs, particularly in settings where conventional therapies (hemodialysis, peritoneal dialysis) are unavailable or inaccessible. Based on the accumulated modeling, bench, and animal evidence, four clinical scenarios currently appear most promising for first translational studies:

Pediatric AKI, where the favorable body-size ratio between a small patient and an adult buddy yields the most efficient urea clearance per session (Section 3.1) and where the treatment duration required (days, not months or years) limits buddy exposure to a defined, self-resolving episode of care. Pediatric AKI may represent a physiologically favorable eventual indication for these reasons, though its appropriateness as the first-in-human population will require independent ethical and regulatory assessment (Section 4.4).

AKI in resource-limited adult and pediatric settings, where conventional dialysis or continuous renal replacement therapy is unavailable due to water, electricity, or equipment constraints, and where even a lower-efficiency but readily deployable modality could meaningfully reduce AKI mortality.

Severe neonatal jaundice (SNJ), as a modeling-stage extension of the same principle (Section 3.6), which, if corroborated by ex vivo and animal studies, could offer an extracorporeal complement to exchange transfusion in settings with a high SNJ burden and limited access to timely, safe exchange transfusion; given the extracorporeal circuit required in a 2.5–3.5 kg neonate, this application cannot yet be considered lower-risk than exchange transfusion or blue-light phototherapy, and remains a modeling-stage concept requiring substantial further validation.

Disaster response and austere or military settings, where AKI incidence rises sharply precisely when conventional HD infrastructure is most likely to be disrupted or entirely absent; a device that eliminates the water-treatment and dialysate-production burden of conventional HD may, in principle, be better matched to these conditions than a conventional dialysis machine dependent on utilities and logistics, though it still requires a healthy volunteer (e.g., in the military setting a comrade of the patient), vascular access, an alloHD machine, infection screening of both participants, and trained personnel. One may want to consider an emergency system akin to the walking blood bank [40].

Chronic maintenance therapy for ESKD appears, on current evidence, to be the more distant and ethically complex application, consistent with the lower stated willingness among caregivers and clinicians to commit to intermittent, indefinite buddy participation relative to a one-time kidney donation (Section 5.2). These scenarios partially overlap with other proposed technologies for dialysis in austere environments [39], such as manual single lumen alternating micro-batch hemodiafiltration (mSLAMB) [41,42].

## 8. Open Questions and a Research Agenda

Several gaps must be addressed before alloHD can be responsibly evaluated in humans. First, vascular access: reliable, adequate flow-capable access must be developed and validated in both patient and buddy, informed by, but not directly transposable from, existing non-CKD arteriovenous access experience. In pediatric and neonatal settings (AKI, SNJ, some metabolic diseases) with low-weight patients, the buddy’s peripheral veins may deliver sufficient blood flow, voiding the need for central access. Second, repeated-exposure buddy safety: the nine-session porcine study is reassuring but limited in duration, species, and sample size; larger-animal studies with longer follow-up, histological assessment of buddy kidneys, and dose–response characterization of buddy solute/fluid loading are needed before extrapolation to chronic human use. Third, blood-leak detection: because alloHD directly opposes two people’s blood across a single membrane, a dedicated, alloHD-specific real-time leak-detection strategy beyond what is required in conventional HD should be engineered and validated. Fourth, buddy logistics and “buddy fatigue and burden”: the sustainability of recurring buddy participation, and its psychological and social dynamics within a patient–buddy dyad, can only be characterized in clinical studies and warrants dedicated qualitative, economic, psychosocial and health-services research. Fifth, dose optimization and solute-specific transport: modeling to date has focused on urea, acid–base parameters, creatinine, and two PBUTs; broader characterization of middle-molecule and drug transport, alongside the bidirectional transfer of beneficial buddy-derived solutes (bicarbonate, water-soluble vitamins, amino acids), remains incomplete. Sixth, regulatory and trial design: as the consulted ethicist noted, progression to human study (e.g., pediatric AKI) will require IRB-approved protocols informed by sufficient animal safety data, and, given alloHD’s novelty, likely benefits from early, coordinated engagement with international nephrology and pediatric nephrology organizations (e.g., the International Society of Nephrology, the International Pediatric Nephrology Associations) to harmonize evidentiary expectations across jurisdictions before first-in-human study. Lastly, country- and region-specific health economic analysis of alloHD has not been performed yet.

## 9. Conclusions

Allo-hemodialysis aims to expand access to affordable KRT performed in resource-limited settings by substituting a healthy human buddy’s blood and intact renal function for the water-treatment and dialysate-production apparatus of a conventional dialysis machine. Two-compartment kinetic modeling indicates that adequate weekly urea clearance and meaningful correction of metabolic acidosis are achievable, provided session frequency is adjusted for the lower per-session efficiency inherent to a physiological, rather than manufactured, dialysate. A low-cost, off-the-shelf prototype has demonstrated the required ultrafiltration accuracy and solute transport ex vivo. Repeated in vivo porcine studies have shown intradialytic reductions in azotemia observed repeatedly within porcine AKI–buddy dyads, with no deterioration detected in the buddy’s measured short-term renal functional parameters. A parallel modeling extension suggests the alloHD principle may be applicable to severe neonatal jaundice. An application of miniaturized alloHD in some life-threatening inborn errors of metabolism as well as certain forms of liver failure are conceivable. Early ethical consultation found no categorical objection to continued research, and caregiver and clinician surveys suggest a meaningful, though not universal, willingness to serve in the buddy role.

Before alloHD can be thoughtfully and responsibly tested in humans, further large-animal and longer-duration safety studies, dedicated vascular-access and leak-detection engineering must be undertaken. Once all preliminary safety issues have been resolved, a prospectively designed, IRB-approved, phase I clinical protocol is needed to test the physical, psychological, social, and financial burdens of serving as an alloHD buddy. Respect for autonomy requires informed, voluntary consent, safeguards against coercion or undue influence, and the right to withdraw at any time without penalty. Withdrawal procedures must allow prompt, safe discontinuation while protecting both participants. Willingness to participate does not, by itself, establish medical eligibility; exclusion or discontinuation criteria should be transparent, proportionate to foreseeable risks, and subject to independent ethical and applicable regulatory oversight. Such safeguards should protect buddies while respecting their autonomy and avoiding unjustified restrictions on participation.

If these further steps raise no critical concerns, alloHD could offer a practical means of addressing the socioeconomic, infrastructural, and workforce constraints that currently, and for the foreseeable future, limit global access to KRT. Rather than representing an incremental optimization of conventional dialysis, alloHD could provide a fundamentally different path forward, particularly for patients with pediatric AKI and for resource-limited populations in whom conventional dialysis technologies remain, and are likely to remain, out of reach. Viewed from this perspective, the ethical question then arises, is it ethical not to pursue this approach to reducing the global health inequity in treating kidney disease? Taken together, we believe the relevant question is no longer only whether alloHD is safe and effective enough to pursue, but whether continued underinvestment in interim approaches such as this, while more comprehensive solutions to the global kidney failure crisis mature, is itself an ethical choice the field should be prepared to defend.

## Figures and Tables

**Table 1:** Resource and complexity comparison between conventional hemodialysis and allo-hemodialysis.

Parameter	Conventional HD	AlloHD
Dialysate source	Manufactured on-site (RO water + concentrate)	Buddy’s own blood
Source water per 4 h session	~300–800 L (with RO reject)	None required
Electricity per session	~7–16 kWh	Minimal (pumps, sensors, tablet)
Core equipment	Water-treatment train, dialysate mixing/heating, multi-sensor console	Dialyzer, 3 blood pumps, EMF balancing sensor, tablet GUI
Approx. machine cost	USD 15,000–25,000 ^1^	Off-the-shelf components; prototype ~1000–2000 USD (estimate) ^2^
Second healthy person required	No	Yes (“buddy”), each session
Bidirectional solute transfers to and from a healthy person	Not applicable	Bicarbonate, vitamins, amino acids diffuse to the patient’s blood; uremic solutes diffuse from patient to the buddy and are cleared by buddy’s kidneys
Treatment time	Usually, 3–4 h per session X 3 per week based on adequacy	Depends on patient/buddy sizes (see text)
Staff	Specialized staff for water system, dialysis machines, patient care, administration	Care partner to connect patient and buddy and operate the alloHD machine

^1^ Manufacturer’s selling price; ^2^ Prototype component cost; not inclusive of regulatory, manufacturing, maintenance, disposables, training, or service costs.

## Data Availability

No new data were created or analyzed in this study. Data sharing is not applicable to this article.

## References

[1] Bello A.K., Levin A., Lunney M., Osman M.A., Ye F., Ashuntantang G.E., Bellorin-Font E., Gharbi M.B., Davison S.N., Ghnaimat M. (2019). Status of care for end stage kidney disease in countries and regions worldwide: International cross sectional survey. BMJ.

[2] Liyanage T., Ninomiya T., Jha V., Neal B., Patrice H.M., Okpechi I., Zhao M.-H., Lv J., Garg A.X., Knight J. (2015). Worldwide access to treatment for end-stage kidney disease: A systematic review. Lancet.

[3] Bello A.K., Okpechi I.G., Levin A., Ye F., Damster S., Arruebo S., Donner J.-A., Caskey F.J., Cho Y., Davids M.R. (2024). An update on the global disparities in kidney disease burden and care across world countries and regions. Lancet Glob. Health.

[4] Wainstein M., Tiv S., Arruebo S., Caskey F.J., Damster S., Donner J.-A., Gouda Z., Jha V., Levin A., Nangaku M. (2025). Global Policy and Advocacy Initiatives for Improving Kidney Care: Report from the 2023 International Society of Nephrology Global Kidney Health Atlas. Kidney360.

[5] Backston K.J., Thompson C.J., Raina M., Sathik N.R., Noh L.S., Lokkur P.P., Bansal S.B. (2025). Green Dialysis Review: Reducing Water, Energy, and Waste. Hemodial. Int..

[6] Ben Hmida M., Mechichi T., Piccoli G.B., Ksibi M. (2023). Water implications in dialysis therapy, threats and opportunities to reduce water consumption: A call for the planet. Kidney Int..

[7] Jimenez M.D.A., Audije-Gil J., Martinez R., Vaquero N.M., Gómez M., Portillo J., Pereda G., Gascueña D.H., Duane B., Sanjuan M. (2025). How to improve the environmental impact in haemodialysis: Small actions, big changes. Clin. Kidney J..

[8] Barraclough K.A., Berman-Parks N., Jha V., Piccoli G.B., Stigant C., Talbot B., Tarrass F., Cheung M., King J.M., Grams M.E. (2026). Green dialysis: Environmentally sustainable care, growth, and innovation: Conclusions from a Kidney Disease: Improving Global Outcomes (KDIGO) Controversies Conference. Kidney Int..

[9] Jiang Y., Zhang S. (2026). Green nephrology: Mitigating the environmental burden of dialysis and promoting sustainable kidney care. Front Med..

[10] Kotanko P., Maheshwari V., Thijssen S. (2018). Allo-hemodialysis: Intermittent donation of kidney function as a novel treatment for patients with kidney failure in limited resource settings. J. Am. Soc. Nephrol..

[11] Kuehn B.M. (2019). Buddy Dialysis Probed as Hemodialysis Alternative. ASN Kidney News.

[12] Kotanko P., Thijssen S. (2019). Virtual Kidney Donation.

[13] Maheshwari V., Grobe N., Wang X., Patel A., Cherif A., Tao X., Chao J., Heide A., Nikolic D., Dong J. (2024). Allo-Hemodialysis, a Novel Dialytic Treatment Option for Patients with Kidney Failure: Outcomes of Mathematical Modelling, Prototyping, and Ex Vivo Testing. Toxins.

[14] Maheshwari V., Thijssen S., Tao X., Fuertinger D., Kappel F., Kotanko P. (2017). A novel mathematical model of protein-bound uremic toxin kinetics during hemodialysis. Sci. Rep..

[15] Maheshwari V., Thijssen S., Tao X., Fuertinger D.H., Kappel F., Kotanko P. (2019). In silico comparison of protein-bound uremic toxin removal by hemodialysis, hemodiafiltration, membrane adsorption, and binding competition. Sci. Rep..

[16] Bankir L., Bouby N., Trinh-Trang-Tan M.M., Ahloulay M., Promeneur D. (1996). Direct and indirect cost of urea excretion. Kidney Int..

[17] Brulles A., Gras J., Magrina N., Torres N., Caralps A. (1969). Relation between urea clearance and glomerular filtration rate according to urine flow-minute. Clin. Chim. Acta.

[18] Cherif A., Maheshwari V., Fuertinger D., Schappacher-Tilp G., Preciado P., Bushinsky D., Thijssen S., Kotanko P. (2020). A mathematical model of the four cardinal acid-base disorders. Math. Biosci. Eng..

[19] de Vries J.C., Bras J.G., de Vries G.M., Vollenbroek J.C., Wieringa F.P., Jankowski J., Verhaar M.C., Stamatialis D., Masereeuw R., Gerritsen K.G.F. (2026). Strategies for Removal of Protein-Bound Uremic Toxins in Hemodialysis. Toxins.

[20] Wang X., Patel A., Gothi A., Nikolic D., Heide A., Dong J., Zhang H., Maheshwari V., Grobe N., Nayak K.S. (2021). Feasibility of Allo-Hemodialysis: First Experience from Porcine Studies. J. Am. Soc. Nephrol..

[21] Wang X., Patel A., Gothi A., Nikolic D., Heide A., Dong J., Maheshwari V., Grobe N., Nayak K.S., Kotanko P. (2022). Allo-Hemodialysis Feasibility Study in a Porcine Model of AKI. J. Am. Soc. Nephrol..

[22] Wang X., Soni P., Patel A., Nikolic D., Heide A., Dong J., Maheshwari V., Grobe N., Nayak S., Kotanko P. (2024). Successful application of allo-hemodialysis in a porcine model of embolization-induced acute kidney injury. Nephrol. Dial. Transplant..

[23] Wang X., Soni P., Patel A., Nikolic D., Heide A., Dong J., Maheshwari V., Grobe N., Nayak K. (2026). Effects of allo-hemodialysis on donor (“buddy”) kidney function in a porcine AKI model. Kidney Int. Rep..

[24] Slusher T.M., Zamora T.G., Appiah D., Stanke J.U., A Strand M., Lee B.W., Richardson S.B., Keating E.M., Siddappa A.M., Olusanya B.O. (2017). Burden of severe neonatal jaundice: A systematic review and meta-analysis. BMJ Paediatr. Open.

[25] Maheshwari V., Diaz-Gonzalez de Ferris M.E., Filler G., Kotanko P. (2024). Novel extracorporeal treatment for severe neonatal jaundice: A mathematical modelling study of allo-hemodialysis. Sci. Rep..

[26] Ashkenazi T., Cohen J., Gelman D., Katvan E. (2023). The psychological examination and evaluation of unrelated kidney donors in Israel: A suggested model. Psychol. Health Med..

[27] Gelderblom H.R., Baron S. (1996). Structure and Classification of Viruses. Medical Microbiology.

[28] Dukhin S.S., Tabani Y., Lai R., Labib O.A., Zydney A.L., Labib M.E. (2014). Outside-In Hemofiltration for Prolonged Operation without Clogging. J. Memb. Sci..

[29] Lenihan C.R., Busque S., Derby G., Blouch K., Myers B.D., Tan J.C. (2015). Longitudinal study of living kidney donor glomerular dynamics after nephrectomy. J. Clin. Invest..

[30] Al-Amin A., Wood J., Atturu G., Gouda M.R., Donnellan C.F., Burke D.A. (2013). Use of arteriovenous fistulae for home parenteral nutrition—A review of the literature. J. Vasc. Access..

[31] Delville M., Manceau S., Ait Abdallah N., Stolba J., Awad S., Damy T., Gellen B., Sabbah L., Debbache K., Audard V. (2017). Arterio-venous fistula for automated red blood cells exchange in patients with sickle cell disease: Complications and outcomes. Am. J. Hematol..

[32] McCarthy W.J., Valentino L.A., Bonilla A.S., Goncharova I., Taylor A., Pooley T.A., Jacobs C.E. (2007). Arteriovenous fistula for long-term venous access for boys with hemophilia. J. Vasc. Surg..

[33] Campos I., Arellano J., Gomez V., Quiroz J., Mariscal L.A. (2020). Renal Replacement Therapy Preferences Survey: Is Allo-Hemodialysis an Acceptable Option for Patient Caregivers and Health Care Professionals?. Blood Purif..

[34] Intas G., Rokana V., Stergiannis P., Chalari E., Anagnostopoulos F. (2020). Burden and Sleeping Disorders of Family Caregivers of Hemodialysis Patients with Chronic Kidney Disease-End Stage: A Cross-Sectional Study. Adv. Exp. Med. Biol..

[35] Solaimanimoghaddam R., Beydokhti T.B., Firouzkohi M.R. (2024). Family Caregivers’ Experiences of Living with Hemodialysis Patients: A Descriptive Phenomenology. Iran. J. Nurs. Midwifery Res..

[36] Skoulatou M., Govina O., Kalemikerakis I., Prountzou A., Tsougia P., Kavga-Paltoglou A. (2024). Burden and Quality of Life of Family Caregivers Caring for Patients on Hemodialysis. Nephrol. Nurs. J..

[37] Fatona E.A. (2026). Strategies to Reduce Caregiver Burden among Family Caregivers of Patients on Hemodialysis. Nephrol. Nurs. J..

[38] Gagel A., Wiesen G., Stuard S., Canaud B. (2026). Advances in Membrane, Dialyzer Design, and Related Monitoring Technologies for Hemodiafiltration: Translating Bench-Side Innovations to Bedside Applications. J. Clin. Med..

[39] Yuan C.M., Perkins R.M. (2011). Renal replacement therapy in austere environments. Int. J. Nephrol..

[40] Brigmon E.P., Cirone J., Harrell K., Greebon L., Ngamsuntikul S., Mendoza A., Epley E., Eastridge B., Nicholson S., Jenkins D.H. (2024). Walking blood bank: A plan to ensure self-sufficiency in an era of blood shortage. Trauma Surg. Acute Care Open.

[41] Lanker S.L., Pino C.J., Humes H.D., Chawla L., Johnston K.A. (2024). In vivo Assessment of a Manual Single Lumen Alternating Micro-Batch Hemodiafiltration System. Blood Purif..

[42] Chawla A.K., Morgan J., Rose J., Ceschia G., Goldstein S.L., Hasson D.C. (2023). Manual Single-Lumen Alternating Micro-Batch Device as Renal Replacement Therapy in Austere Environments. Blood Purif..

